# The Effect of Periodic Loading of Glued Laminated Beams on Their Static Bending Strength

**DOI:** 10.3390/ma15113928

**Published:** 2022-05-31

**Authors:** Dorota Dziurka, Adam Derkowski, Dorota Dukarska, Jakub Kawalerczyk, Radosław Mirski

**Affiliations:** Department of Mechanical Wood Technology, Poznań University of Life Sciences, Wojska Polskiego 28, 60-627 Poznań, Poland; adam.derkowski@up.poznan.pl (A.D.); dorota.dukarska@up.poznan.pl (D.D.); jakub.kawalerczyk@up.poznan.pl (J.K.); radoslaw.mirski@up.poznan.pl (R.M.)

**Keywords:** glulam beams, mechanical properties, periodic loading, beams, glued laminated timber

## Abstract

Engineered wood products (EWP) such as glulam beams are gaining more and more popularity due to several advantages resulting from the wood itself, as well as the constant search for structural materials of natural origin. However, building materials face some requirements regarding their strength. Thus, the study aimed to assess the static bending strength of structural beams produced with the use of pine wood, after the periodic loading of approximately 80 kN for a year. The manufactured beams differed in the type of facing layers, i.e., pine timber with a high modulus of elasticity and plywood. The produced beams, regardless of their structure, are characterized by a similar static bending strength. Moreover, it has been shown that the loading of beams in the range of about 45% of their immediate capacity does not significantly affect their static bending strength and linear modulus of elasticity.

## 1. Introduction

Wood is generally a very durable material, and resistant to many aggressive both biotic and abiotic factors after proper treatment. Well-prepared structures can survive even several centuries, and the oldest objects made of wood are approaching 1000 years of age. Examples of this can be found in Japan or Norway, where sacred monuments come from the 8th and 11th centuries. Because wooden structures are still a vast and essential part of construction, even traditional brick construction, the condition and performance of structural elements in many countries are essential to ensure the safety of their use. Due to the characteristics (on the one hand, natural; on the other hand, perceived as technical disadvantages), wood is more troublesome due to the anatomical defects, anisotropy, and less homogeneity than steel or concrete. However, for the so-called sustainable development [1,2], a building currently has no better material than wood [3,4]. Compared to steel and concrete structures, wood structures have a smaller carbon footprint [5,6]. Thus, there is now a worldwide resurgence in this type of construction [7,8]. Some circles even believe that the 21st century is destined to be the century of timber construction. Since the availability of solid wood structural members is limited by the size of the roundwood harvested and the distribution of defects in that wood, it is more advantageous to use glued laminated timber (GL, glulam) (EN 14080 [9]). Structural elements of this type can be produced without considering the dimensions of the harvested wood or the distribution of defects on its cross-section [10,11,12]. However, assessing the quality of each lamella allows for better shaping of the future beam. It is advantageous to stack higher quality pieces in more stressed areas [13,14,15]. Glulam has the typical characteristics of solid wood: lightness, good strength, flexibility, durability, and ease of processing. However, it is characterized by its ease of forming into sections compared to wood. The lumber is glued with binders that guarantee high strength under static and dynamic loads. Glued laminated timber, especially that intended for load-bearing structures, is almost always glued with resorcinol-phenol-formaldehyde (PRF) or melamine-urea-formaldehyde (MUF) resin [16,17,18]. In addition, a polyurethane-based adhesive is becoming more popular. These adhesives should provide high durability under varying environmental conditions [19,20,21]. Therefore, it is assumed that it is the wood that will deteriorate faster than the glue joint. However, a poorly made joint or an improperly selected adhesive for the conditions can cause the beam to delaminate [22,23]. A delaminated beam no longer has the same load capacity as a whole/primary beam. Beams made of glued laminated lumber also show great potential for modifying their construction, essentially strengthening them using different non-wood materials. The most common are steel [24,25,26,27], various types of fiber components or mats [28,29,30,31,32], and concrete [33,34,35,36]. These modifications result in increased beam stiffness, strength, and fire resistance. The increase in stiffness or load carrying capacity seems to be the most relevant since the fire resistance is partly related to the cross-section used.

Our previous studies showed that beams made of non-defective pine lumber, immediately after manufacturing, exhibit good technical properties (high static bending strength and modulus of elasticity) [18]. It has also been shown that it is possible to produce beams whose face layer (outer lamella of the tension zone) is made of plywood or LVL. This makes it possible to produce glulam beams of high quality in the absence of lumber of a quality that allows it to be used for this layer. However, the structural elements in buildings are usually subjected to a constant load over time throughout their service life. The initial values of these loads are known at the beginning, and at the construction design stage. Because the materials with different elastic properties are used, including plywood, it is important to investigate how such variants will perform after a longer period of loading. For these reasons, the paper evaluates the effect of the loading time of the structure of both types of manufactured beams on their quality after load removal.

## 2. Materials and Methods

The beams were made of thick pine timber or pine timber combined with one outer layer of plywood. Experimental timber had the following dimensions: 137 mm wide × 40 mm thick × 3485 long. The timber pieces were obtained from logs originating from the Forest Division Olesno (50°52′30″ N 18°25′00″ E). Plywood 21 mm thick (Biaform S.A., Bialystok, Poland) was used in this study and its characteristics were given in an earlier publication [37]. In addition, previous work [18,38,39] suggests that it is more advantageous to sort lumber visually and determine its linear elastic modulus sonically or mechanically, and then form beams with the assumption that the closer to the center of the designed beam, the lower quality lumber can be used. For these reasons, beams fabricated from lumber graded according to the linear modulus of elasticity as determined by the 4-point bending test were subjected to the same test. These beams were produced as 8-layer beams, assuming that the position of the lamella in the beam, except for the outer layer/tension lamella, was determined by its modulus of elasticity. In the case of the face layer, the number and distribution of knots are also important. It is assumed that there must be no rotten knots or knotholes in the edge zone. The face layers had a very high modulus of elasticity for these beams, at about 15 GPa. The individual lamellas were tested in terms of a modulus of elasticity according to the method presented in the previous study [27]. On the other hand, the middle layer was a low-quality sawn timber and would be classified as C16 according to EN 338 [39]. On the larger/wider surface of the board, there may be healthy knots occupying not more than half of the cross-section or broken knots up to one-third of the cross-section. Lumber with this type of defect distribution, regardless of the modulus of elasticity, was laid in layers closer to the neutral axis. 

**Figure 1:**
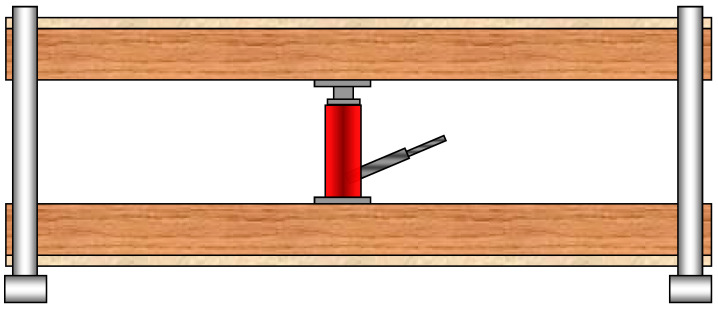
Beam attachment system in the yokes.

The beams were manufactured in a standard manner for the project. Melamine-urea-formaldehyde adhesive (MUF 1247) purchased from AkzoNobel (Amsterdam, The Netherlands) was applied on the surface of the lumber in the amount of 220–240 g/m^2^ as recommended by the producer, mixed with a dedicated hardener labeled as 2526 in the amount of 10% of the dry mass of MUF resin. The press loading process took up to 20–22 min. After loading, the press was closed, and the proper press pressure of about 0.48–0.50 MPa was exerted. The press was closed (pressure was exerted) for at least 4 h. Some of the produced beams were examined after the conditioning period, while the remaining 8 were placed in yokes (Figure 1), and the pressure value in the hydraulic cylinders was controlled for more than one year. 

The applied load was assumed to be 0.55–0.6 of the average beam failure force in the 4-point bending test, i.e., between 80 and 85 kN (8–8.5 tons). The yokes with the beams were set up in the laboratory hall, which is a heated hall with an average temperature of about 20 °C during the winter. After the bending load period, the beams were subjected to the strength and modulus of elasticity investigations in a 4-point bending test. The detailed description of the test method and the equipment used can be found in our previous work on structural beams [18,40]. The results obtained were statistically analyzed and related to the results of previous studies. Statistica version 13.0 (Version 13.0, StatSoft Inc., Tulsa, OK, USA) was used for statistical evaluation.

The following test designations were introduced:

- A—beams with a plywood face not subjected to the long-term loading process;
- B—beams with a plywood face in the long-term loading process;
- P—beams with the face of visually graded lumber not subjected to long-term loading;
- Z—beams with the face of visually graded lumber subjected to long-term loading.

## 3. Results and Discussion

The mechanical properties of the layers used (averaged values of the lumber used) for the beams containing plywood are shown in Table 1. As can be seen from the data presented there, although the elastic modulus of plywood is lower than that of lumber, its strength is several times higher than that of lumber. Table 2, on the other hand, shows the arrangement of lamellas in beams manufactured from pine lumber only. 

When laying out the sets of lumber for future beams, we use higher and lower quality lumber. The latter is not usually used as structural lumber for essential loading elements. However, we obtain elements with a high carrying capacity by using them as a beam components. The expected modulus of elasticity of produced beams should be about 13.7 kN/mm^2^ in the case of beams made of pine lumber only, and about 10.7 kN/mm^2^ in the case of beams containing a plywood face. 

**Figure 2:**
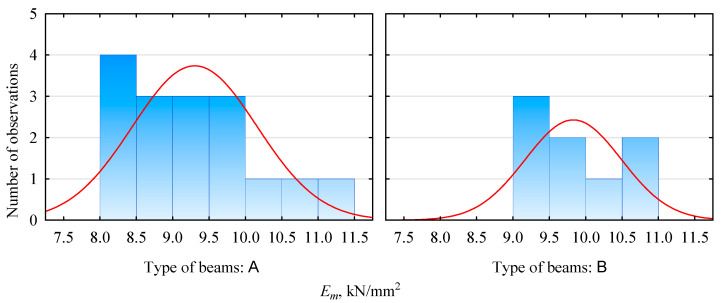
Modulus of elasticity distribution for beams manufactured with plywood face.

**Figure 3:**
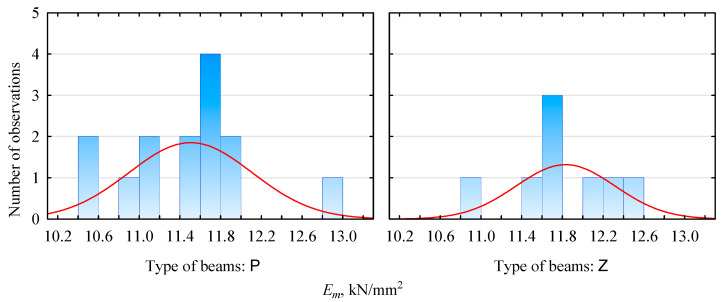
Modulus of elasticity distribution for beams manufactured with the face of visually graded lumber.

The modulus of elasticity evaluation results is presented in Table 3 and Figure 2 and Figure 3. The produced beams, irrespective of the storage method, show similar values in terms of the average modulus of elasticity. It is in the range of 11.5–11.8 kN/mm^2^ for pine beams and 9.3–9.8 kN/mm^2^ for beams with plywood faces. In both cases, it is lower than expected. However, this may be due to the specificity of the determination of the modulus of elasticity of the lamellas. Those values do not directly translate to the modulus of elasticity of the beams. However, very importantly, there is no statistical basis for both batches to reject the null hypothesis that there is no effect of exerting an external load for one year on the modulus of elasticity. In other words, we do not reject the null hypothesis that the elastic modulus of the two batches (before and after the conditioning period) is equal.

Furthermore, as can be seen from the data presented in Table 3, the standard deviations for the two samples are not similar and are slightly higher for the unconditioned samples. The lower values of the standard deviation determined for the aged samples may indicate, on the one hand, a greater stabilization of the beam structure. Still, on the other hand, they may indicate only the quality of the selected batch of lumber for the test sample, since the analyzed sets are not very large. The distribution shown in Figure 3 shows that some differences may be due only to differences in the sizes of the test samples, since the basic unit sizes are represented, if they are already, by a similar number of samples.

A similar behavior of loaded and unloaded beams is observed in terms of static bending strength. Again, the average strength of the analyzed beams evaluated after the testing period is similar to that of the assessed beams immediately after manufacturing. Thus, the static flexural strength for beams manufactured with a plywood face immediately after manufacturing (variant A) is 42.8 N/mm^2^, and after applying a constant force, 44.5 N/mm^2^. For beams made of pine lumber only, the static flexural strength immediately after fabrication is 42.6 N/mm^2^, and after the evaluation period, 41.0 N/m^2^. Thus, the relative differences in the two types of beams are the same, i.e., approximately ±1.7 N/mm^2^. However, there is no basis to reject the null hypothesis that the flexural strengths of the beams are equal, regardless of their strength evaluation period (Table 4).

**Figure 4:**
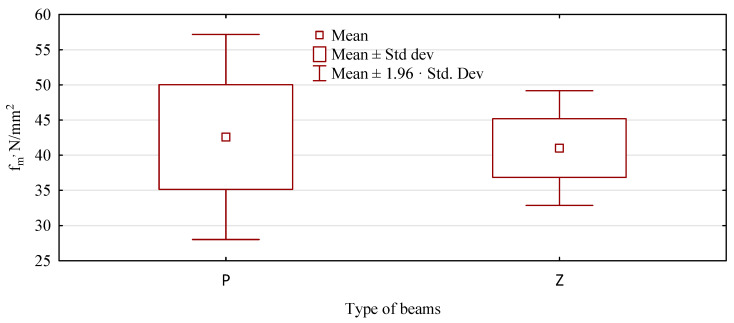
Static bending strength of pine beams.

The data shown in Figure 4 indicate that primary beams (not loaded) have a much more significant variation in static flexural strength than beams subjected to long-term loading. In the case of type A and B beams, we have the opposite situation. Thus, the variability (amount of scattering), or lack thereof, should not only be attributed to the testing process as an effect of long-term loading but primarily to the variability of the raw material. Both high and very low values of the static bending strength are mainly related to the quality of the given piece of lumber and its position in the beam. This can be seen more clearly in beams made from pine lumber alone.

**Figure 5:**
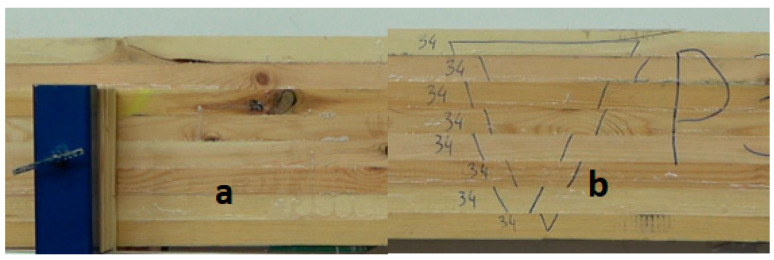

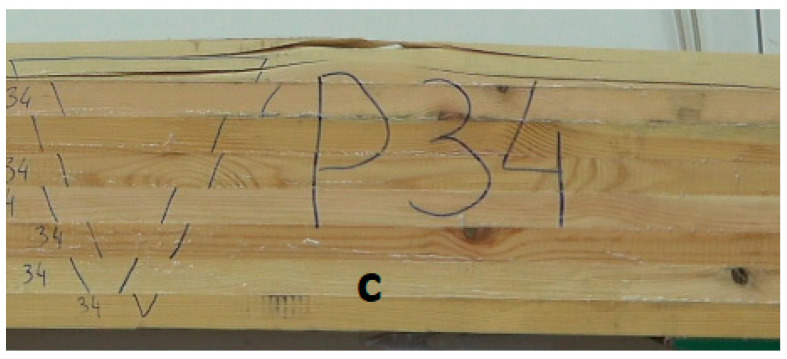
Fracture process of a beam manufactured from pine lumber.

The quality of the manufactured beam is determined by human error, and only in the next case by invisible material characteristics. Figure 5 shows the failure points of the lumber/outer lamellae, which cause the loss of load-carrying capacity of the tested beam. The cracking/destruction process of the beam starts from location a (Figure 5—position a). On the plane of the beam at this location, a located twist/deformation of the fibers can be seen, indicating that there was or is a knot nearby. However, there is a crack: delamination of the lumber at another location (Figure 5—position b). Initially, the cracks propagate to the left from this area and later to the right, eventually leading to the complete failure of the beam (Figure 5—position c). 

**Figure 6:**
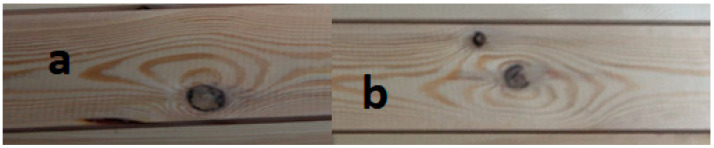
The appearance of critical areas of lumber forming the face of P34 beam—side from lamella 2.

Since all pieces of lumber are surface scanned before gluing, a more extensive analysis of the failure process of beam P34 can be made. However, the first crack, which is not the cause of the beam failure, occurs at the location a, which is the location of a mortise knot about 1/4–1/3 of the board width (138 mm) in the plane shown in Figure 6. The knot is located approximately 10 mm from the edge. 

There are two little broken knots in the damaged area (Figure 6—position b): one in the central part of the lumber and the other about 7–10 mm from the edge. Thus, the appearance of these sites does not deviate from the assumptions. Since these are the worst places on the surface of the used piece, they should and ultimately are the places where the destruction of the beam began.

**Figure 7:**
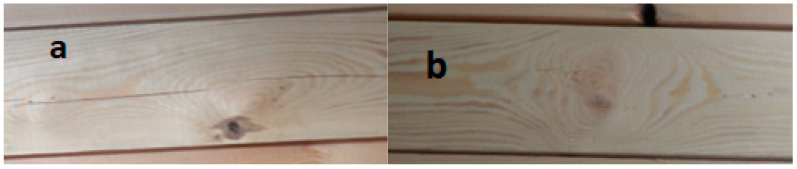
The appearance of critical areas of lumber forming the face of the P34 beam—exterior side of the beam.

The other side of the lumber used (Figure 7), which is the face of the beam, looks much better. There is a small rotten knot in zone a, about 15 mm long. However, in the middle part of the lumber, in zone b, a significant fiber twist and only one knot, closer to the axis of the board, are visible. Based on this, the lumber could be considered good quality, and the failure would occur in the tension lumber, in the middle zone (between thrusts), where the highest value of bending modulus occurs.

**Figure 8:**
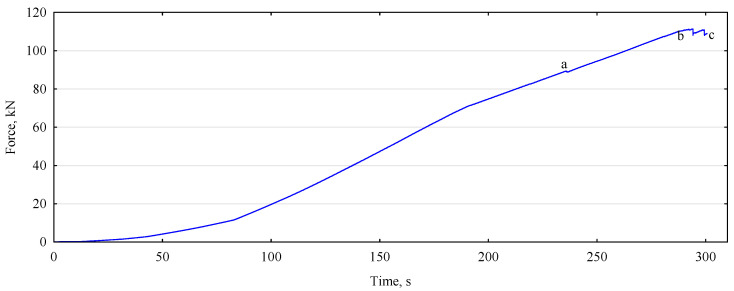
Process of loading the P34 beam to failure.

The analyzed beam achieves a strength of 30.6 N/mm^2^, slightly higher than assumed for this set of beams (30 N/mm^2^ was taken). The maximum failure force for this beam is approximately 111 kN (Figure 8). However, the first area of the instantaneous force drop, associated with cracking the beam at the location a, occurs already at a load of 89.5 kN. It corresponds to a strength of less than 25 N/mm^2^. However, the beam does not crack. Additionally, after a moment, there is an increase in the loading force, lasting about 60 s. During this time, the force increases further by about 25% of point a, its maximum value. A crack occurs at point b. This delamination is followed by a further increase in the force (position c) to a value close to the maximum (value lower by about 0.3 kN). Only then, does the beam’s complete, although slow, destruction occur. The distribution of defects in tension lumber is crucial in designing beams made from non-defective lumber. This assessment must be made at each plane of the lumber to avoid incorrect assessments of the piece’s quality. 

One of the crucial characteristics of wood, and therefore of structural beams made of wood, is the dependence of specific mechanical properties on moisture content. Although there is an ongoing debate as to how important these changes are, and whether a given mechanical quantity determined under given conditions should ultimately be converted to another, in the field of the influence of moisture content on the modulus of elasticity, this discussion is “least of all”. Most researchers agree that even within the range of usable moisture contents, the obtained values of an elastic modulus at a given moisture content should be converted to a “moisture content” of 12%. Therefore, to compare different batches of wood or wood elements in the simplest, correct way, it is best to determine their properties at similar moisture contents. The statistical analysis, the results of which are presented in Table 5, shows no grounds for concluding that the moisture contents of the two batches of beams/test samples are statistically different.

**Figure 9:**
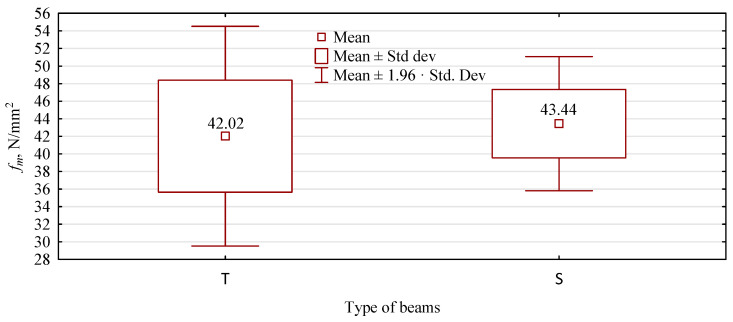
Static bending strength of analyzed beam types.

As there are no significant differences between the evaluation before and after the loading period, both variants are treated as one, and beams made with a plywood face (T) are evaluated in a larger sample against beams made only of pine lumber (S). Both types have similar static bending strengths (Figure 9). T-type beams show an average bending strength of 42 N/mm^2^ and beams with a plywood face, 43.4 N/mm^2^. A difference of approximately 1.4 N/mm^2^ is not statistically significant (*p* = 0.3535) and is even less technologically substantial.

However, the strength range represented by the whiskers in Figure 9 for beams containing plywood is much smaller and represents only about 60% of the strength range for beams made from pine lumber alone. The assumed range of whiskers is as high as the 95% confidence interval, thus indicating that 95% of the strength of a beam manufactured according to the described procedure will fall within this range. Significant differences, statistically significant in terms of the spread of values around the mean value, are also evidenced by Levene’s test analysis of the homogeneity of variance. The obtained statistic values make it necessary to reject the hypothesis of an equality of variances (Table 6). Thus, beams made with a plywood face are more homogeneous in strength, which may be due to the uniform quality of the plywood throughout the sheet. This is undoubtedly a significant advantage. It should also be remembered that the beams made with a plywood face are made from lower quality lumber, assuming a high relationship between the modulus of elasticity of the lumber and the strength of the beam.

## 4. Conclusions

The beams produced, irrespective of their construction, are characterized by similar static bending strengths:

- Beams manufactured with a plywood face have a narrower confidence interval than beams manufactured with pine lumber alone;
- One year of loading in the range of about 45% of the load capacity of the beams did not significantly affect their bending strength or modulus of elasticity;
- The lack of significant changes in the quality of the beams analyzed may be due to the too-short testing period resulting from the duration of the project’s research phase or the level of stress exerted being too low. It seems that the designed structures are rarely loaded continuously with a load of more than 50% of the ad hoc strength. However, the presented results and applied load will be used as an indication for further studies regarding the effect of changing conditions. Taking into account the high impact of humidity which we expect, this study will allow one to compare the results more clearly;
- The presented results could be a valuable reference for designing the next studies. They will help to assume the minimum value of the load.

## Figures and Tables

**Table 1:** Material properties for beams containing plywood face.

Property	Layer							
	Plywood	Lumber 1	Lumber 2	Lumber 3	Lumber 4	Lumber 5	Lumber 6	Lumber 7
	(N/mm^2^)							
*fm*__II_	73.8 *	28.79 ***	24.96	18.87	18.94	24.69	28.96	23.50
*fm*__⊥_	63.3	-	-	-	-	-	-	-
*Em*_II_	8810/8640 **	11,830	10,890	9110	9100	10,820	11,870	10,500
*Em*_⊥_	7840	-	-	-	-	-	-	-

*—Average values for the layer. **—value on small samples/value on large samples. ***—values estimated according to PN-EN 338. II—along fibers of the outer layer. ⊥—perpendicular to fibers of the outer layer.

**Table 2:** Material properties for 8-layer beams.

Property	Layer							
	Lumber 1	Lumber 2	Lumber 3	Lumber 4	Lumber 5	Lumber 6	Lumber 7	Lumber 8
	(N/mm^2^)							
*fm*	44.20	34.16	26.53	14.30	14.36	24.69	32.03	43.74
*Em*	15,130	12,730	11,110	8590	8770	10,820	12,440	14,750

**Table 3:** Statistical analysis of changes in the modulus of elasticity.

Mean Value kN/mm^2^	Mean Value kN/mm^2^	*df*	*t*	*p*	Standard Deviation A/P	Standard Deviation B/Z	*F*-Value Variances	*p* Variances
A	B	Relation A: B						
9.29	9.82	22	−1.519	0.142996	0.854	0.657	1.68854	0.49452
P	Z	Relation P: Z						
11.50	11.82	20	−1.296	0.20960	0.603	0.484	1.55082	0.57418

**Table 4:** Statistical analysis of changes in bending strength.

Mean Value	Mean Value	*df*	*t*	*p*	Standard Deviation	Standard Deviation	*F*-Value Variances	*p* Variances
N/mm^2^	N/mm^2^	-	-	-	N/mm^2^	N/mm^2^	-	-
A	B	Relation A: B			A	B	Relation A: B	
42.8	44.5	22	−0.9975	0.3294	3.887	4.148	1.1385	0.7820
P	Z	Relation P: Z			P	Z	Relation P: Z	
42.6	41.0	20	0.5496	0.5887	7.440	4.164	3.192	0.1300

**Table 5:** Statistical analysis of moisture content in tested pine beams.

Mean Value	Mean Value	*df*	*t*	*p*	Standard Deviation	Standard Deviation	*F*-Value Variances	*p* Variances
%	%	-	-	-	%	%	-	-
A	B	Relation A: B			A	B	Relation A: B	
11.20	12.16	1.876	22	0.7394	1.23888	1.04037	1.41786	0.6634
P	Z	Relation P: Z			P	Z	Relation P: Z	
10.67	10.80	−0.2004	20	0.84317	1.5026	1.0922	1.8928	0.40323

**Table 6:** Homogeneity of variance test for bending strength.

Levene’a F(1.df)	*df* Levene’a	*p* Levene’a
6.47052625	45	0.0144725817

## Data Availability

The data presented in this study are available on request from the corresponding author.
